# Combination of modified albumin-bilirubin grade and platelet count to predict high-risk varices in patients with hepatocellular carcinoma

**DOI:** 10.1371/journal.pone.0327967

**Published:** 2025-07-17

**Authors:** Prooksa Ananchuensook, Kingkomon Piyawannasuth, Sirinporn Suksawatamnuay, Panarat Thaimai, Nipaporn Siripon, Supachaya Sriphoosanaphan, Kessarin Thanapirom, Piyawat Komolmit

**Affiliations:** 1 Division of Gastroenterology, Department of Medicine, Faculty of Medicine, Chulalongkorn University, Bangkok, Thailand; 2 Center of Excellence in Liver Diseases, King Chulalongkorn Memorial Hospital, Thai Red Cross Society, Bangkok, Thailand; 3 Center of Excellence in Hepatic Fibrosis and Cirrhosis, Faculty of Medicine, Chulalongkorn University, Bangkok, Thailand; 4 Academic Affair, Faculty of Medicine, Chulalongkorn University, Bangkok, Thailand; 5 Academic Affair and Division of Gastroenterology, Department of Medicine, Faculty of Medicine, Chulalongkorn University, Bangkok, Thailand; Harvard Medical School, UNITED STATES OF AMERICA

## Abstract

**Background:**

Variceal bleeding is associated with poor prognosis in patients with hepatocellular carcinoma. Therefore, it is essential to identify indicators of high-risk varices (HRV) and provide prompt intervention.

**Aim:**

To validate and modify albumin-bilirubin and platelet scores to predict high-risk varices in patients with hepatocellular carcinoma.

**Methods:**

We enrolled patients with hepatocellular carcinoma and esophagogastroduodenoscopy reports at King Chulalongkorn Memorial Hospital, Bangkok, Thailand, between 2015 and 2022. The nearest demographic and clinical characteristics and laboratory values were reviewed retrospectively within 6 months before the esophagogastroduodenoscopy. Albumin-bilirubin and platelet counts were calculated from the albumin-bilirubin grade plus score from platelet count. We evaluated the new modified albumin-bilirubin and platelet (mALBI-PLT)‘s in predicting HRV by dividing participants into a training cohort (first half) and a validation cohort (second half).

**Results:**

Of 564 patients with hepatocellular carcinoma, 277 were included. Most patients (232 [83.8%]) had Child-Turcotte-Pugh A cirrhosis, whereas 131 (47.3%), 85 (30.7%), and 60 (22.0%) had Barcelona Clinic Liver Cancer stages A, B, and C, respectively. Thirty-eight (15.6%) participants had HRV on esophagogastroduodenoscopy. On multivariate analysis, modified albumin-bilirubin grades 2b and 3 and platelet count ≤150,000/µL were significantly associated with HRV.

With a cut-off value of 2, the mALBI-PLT showed comparable performance in both cohorts, yielding sensitivities of 93.8% and 95.5%, and negative predictive values (NPV) of 98.1% and 98.0%, respectively. In the entire cohort, the albumin-bilirubin and platelet scores, and mALBI-PLT score >2, demonstrated excellent sensitivities (97.4% and 94.7%), respectively, (with NPV of 98.7% and 98.0%), in predicting HRV.

**Conclusion:**

Modified albumin-bilirubin grade and platelet at cut-off 150,000/µL exhibited significant association with high-risk varices in patients with hepatocellular carcinoma. Moreover, hepatocellular carcinoma patients with modified albumin-bilirubin grade 1 or 2a, together with platelets > 150,000/µL, may be able to avoid oesophagogastroduodenoscopy.

## Introduction

Complications, such as portal hypertension (PHT) and variceal bleeding, can occur in patients with hepatocellular carcinoma (HCC) [1]. Factors including HCC, portal vein thrombosis (PVT), and an emerging combination of an anti-vascular endothelial growth factor agent and immunotherapy increase portal pressure and the risk of variceal bleeding [2–4]. Previous studies reported esophageal varices (EV) in approximately 60% of patients with HCC, and demonstrated that those with EV experience shorter survival and higher mortality from variceal bleeding [5,6]. High-risk varices (HRV), defined as varices larger than 5 mm, and large varices (F2 and F3) have a higher chance of bleeding [7]. Primary prevention, either with beta-blockers or esophageal variceal band ligation (EVL), reduces the risk of variceal bleeding and significantly improves the survival of patients with HCC and HRV [8]. Therefore, detecting HRV in patients with HCC is essential for determining the prognosis and providing prompt intervention.

With the introduction of the Baveno VII guidelines, non-invasive tests (NITs) focusing on liver stiffness measurement (LSM) with transient elastography (TE) have replaced the use of the hepatic venous pressure gradient in detecting PHT among patients with cirrhosis [9,10]. However, NITs for patients with HCC still require further validation [1]. In cirrhosis patients, TE ≤ 20 kPa and platelet > 150,000/µL have been established as a cut-off to avoid EV surveillance with esophagogastroduodenoscopy (EGD), according to the Baveno VII consensus [10]. Wu et al. validated this criterion and discovered that less than 5% of patients with HCC within the Baveno VII criteria had HRV, whereas 8% was reported by Allaire et al. [11,12]. As the tumor may falsely increase LSM, the Baveno VII criteria may not apply to patients with HCC, and other NITs or scoring systems to predict HRV need to be investigated [1].

The albumin-bilirubin (ALBI) score predicts survival in patients with HCC and is recognized by the Barcelona Clinical Liver Cancer (BCLC) 2022 guidelines as a prognostic tool [13–15]. Chen et al. explored and validated the combination of the ALBI score and patient platelet counts, referred to as the ALBI-platelet (ALBI-PLT) score, a tool for predicting HRV in patients with HCC [16]. ALBI-PLT, calculated from ALBI grade plus points from platelet count (1 point for > 150,000/µL and 2 points for ≤ 150,000/µL), at a cut-off value of 2, demonstrated an excellent negative predictive value (NPV) of 98.1% [16]. Further studies verified ALBI-PLT in patients with cirrhosis but not in those with HCC [17,18].

Owing to advancements in HCC treatment, the overall survival of patients with HCC has significantly improved over the past decade [13]. Therefore, identifying patients who require HRV surveillance and providing prompt primary variceal bleeding prophylaxis to prevent potential interruption of HCC treatment and improve patient survival are crucial [1,8–10]. Therefore, in this study, we aimed to uncover factors linked to HRV and validate, as well as refine, the ALBI-PLT score as a predictor of HRV in patients with HCC.

## Materials and methods

### Participants and study design

Data from patients diagnosed with HCC at the hepatoma clinic of King Chulalongkorn Memorial Hospital, a tertiary referral center and academic teaching hospital in Bangkok, Thailand, between 2015 and December 2022 were retrospectively reviewed. Data were collected from electronic medical records between September 18, 2023, and November 29, 2023. During the data collection process, the authors had access to information that could potentially identify individual participants. However, all identifying information was removed from the dataset prior to analysis and publication. This project received approval from the Ethics Committee and Institutional Review Board (IRB) of the Faculty of Medicine, Chulalongkorn University, Bangkok, Thailand (IRB Number: 0589/66), and strict confidentiality protocols were maintained throughout the study.

According to the American Association for the Study of Liver Disease guidelines, HCC is diagnosed histopathologically or by a compatible arterial enhancing liver mass with rapid venous washout on contrast-enhanced abdominal imaging in patients with chronic liver disease or cirrhosis [19]. Patients with HCC with available EGD results were enrolled in the study. Patients with the first presentation of upper gastrointestinal bleeding, prior endoscopic intervention, including variceal treatment, and HCC BCLC stage D were excluded.

Baseline characteristics, including age, sex, etiology of liver disease, Child-Turcotte-Pugh (CTP) score, and splenomegaly, (defined by a spleen size > 11 cm on imaging), were recorded. HCC details, including BCLC staging, presence of PVT, and treatment modalities, were collected. Liver function test results were retrospectively documented within 6 months before EGD. Outpatient laboratory values closest to the EGD data and taken at least three months from hospital admission were used to represent the patient’s baseline liver function.

The formula for the ALBI score was 0.66 × log_10_bilirubin (µmol/L) – 0.0085 × albumin (g/L). The ALBI score was categorized as grades 1, 2, and 3, corresponding to ≤ –2.6, > –2.6 to ≥ –1.39, and> –1.39, respectively. Per the study by Chen et al., the ALBI grade and platelet count (ALBI-PLT) was computed by adding the ALBI grade and points for platelet count (1 point for > 150,000/µL and 2 points for ≤ 150,000/µL) [16].

Modified ALBI (mALBI) grade enhances hepatic reserve assessment by subdividing ALBI grade 2 [20–22]. The mALBI grade utilizes the same formula as the ALBI score but was stratified into grades 1, 2a, 2b, and 3 with ≤ –2.6, > –2.6 to ≥ –2.27, > –2.27 to ≥ –1.39 and> –1.39, respectively [20–22]. Therefore, we included mALBI grade in predicting HRV and proposed the modified ALBI-PLT (mALBI-PLT), which was generated from beta-coefficients in the multivariable logistic regression that included ALBI grade and platelet count in the model. Points were scored as follows: mALBI grading has 1 point for grade 1 or 2a, and 2 points for grade 2b or 3, and platelet count has 1 point for > 150,000/µL, and 2 points for ≤ 150,000/µL.

EGD findings were reviewed from electronic medical records. In cases in which multiple EGDs were performed, we collected the first report on the outcome of our study. EVs were divided into three grades: F1, F2, and F3. F1 is a straight varix, F2 is an enlarged or nodular varix occupying less than one-third of the lumen, and F3 is an enlarged or tumorous varix occupying more than one-third of the lumen (F0 refers to normal or no EV) [23]. We classified F2 and F3 EV as HRV that required intervention, including EVL or beta-blockers.

Overall survival was calculated from the date of EGD to the date of death recorded in the Thailand National Death Register. Patients who survived were censored during the most recent follow-up visit.

### Statistical analysis

The SPSS software (version 29.01; IBM Corp., NY, USA) and STATA software (version 18; Stata Corp. LLC, TX, USA) were used for all statistical analyses. Baseline tumor characteristics and laboratory values were presented as frequency (percentage) for categorical variables and mean and standard deviation (SD) for continuous variables. Categorical variables were compared using the chi-square or Fisher’s exact tests, whereas continuous variables were assessed using the Mann–Whitney U test. We evaluated the new modified albumin-bilirubin and platelet (mALBI-PLT)’s in predicting HRV by dividing participants into a 50:50 training and a validation cohorts based on EGD date. The training cohort consisted of participants enrolled from the beginning of the study, and the last participants underwent EGD in December 2018, while the validation cohort consisted of participants who underwent EGD from 2019 to 2022. Additionally, we internally validated the model’s performance with bootstrap resampling method [24].

We then validated the ALBI score across the entire cohort and compared it to the mALBI score. The ability of ALBI and platelet count to predict outcomes was demonstrated using time-dependent receiver operating characteristic analysis. The sensitivity, specificity, negative predictive value, positive predictive value, and both positive and negative likelihood ratios for each score were estimated. Receiver operating characteristic (ROC) analyses were used to compare the ALBI-PLT and mALBI-PLT scores. Logistic regression analysis was used to assess factors associated with HRV, while factors related to overall survival were evaluated using the Cox regression model. Factors with p < 0.05 in the univariate analysis were included in the multivariable model.

## Results

### Demographic, clinical, and tumor characteristics and laboratory values of enrolled patients

Of the 564 patients with HCC, 277 with EGD results were included (S1 Fig). The median follow-up time was 36 months (interquartile range [IQR]: 15.5–54 months). The mean age of the participants was 66.4 years, and 215 (77.6%) were men. Most patients in our cohort had CTP-A cirrhosis 232 (83.8%), and viral hepatitis (78.7%) was the predominant cause of chronic liver disease. Considering the HCC status, 131 (47.3%), 85 (30.7%), and 61 (22.0%) patients had HCC stages BCLC A, B, and C, respectively. Among patients with HCC BCLC C, 31 (50.8%) had PVT.

The median duration between laboratory evaluation and EGD was 13 days (IQR 4–28.5). Table 1 presents the liver function test results and platelet counts of all patients in the cohort. Of these patients, 120 (43.3%), 48 (17.3%), 94 (33.9%), and 15 (5.4%) had mALBI grades 1, 2a, 2b, and 3, respectively.

**Table 1:** Demographic, clinical, and tumour characteristics and laboratory values of all patients and comparison between patients with and without high-risk varices.

Characteristics Number (%)/ mean ± SD	All patients (N = 277)	No HRV (N= 239)	HRV (N=38)
Male	215 (77.6%)	185 (77.4%)	30 (78.9%)
Age	66.42 ± 11.28	66.46 ± 11.63	66.18 ± 8.90
Etiologies of cirrhosis			
HBV	134 (48.4%)	118 (49.4%)	16 (42.1%)
HCV	84 (30.3%)	74 (31%)	10 (26.3%)
HBV and HCV	2 (0.7%)	2 (0.8%)	0 (0%)
Alcohol	27 (9.7%)	23 (9.6%)	4 (10.5%)
MASLD	11 (4.0%)	9 (3.8%)	2 (5.3%)
Cryptogenic	18 (6.5%)	12 (5.0%)	6 (15.8%)
PBC	1 (4.0%)	1 (0.4%)	0 (0%)
Cirrhosis	262 (94.6%)	224 (93.7%)	38 (100%)
CTP			
A (5-6)	232/262 (88.5%)	202/224 (90.2%)	30/38 (78.9%)
B (7-8)	30/262 (11.5%)	22/224 (9.8%)	8/38 (21.1%)
Splenomegaly	141 (54.2%)	110 (46.0%)	31 (81.6%)
BCLC staging			
A	131 (47.3%)	114 (47.7%)	17 (44.7%)
B	85 (30.7%)	71 (29.7%)	14 (36.8%)
C	61 (22.0%)	54 (22.6%)	7 (18.4%)
- PVT	- 31/61 (50.8%)	- 26/54 (48.1%)	- 5/7 (71.4%)
Viable HCC			
Yes	185 (66.8%)	157 (65.7%)	28 (73.7%)
- New case	- 99/185 (53.5%)		
No	92 (33.2%)	82 (34.3%)	10 (26.3%)
Liver function tests			
Albumin (g/dL)	3.73 ± 0.55	3.79 ± 0.54	3.36 ± 0.48
Total bilirubin (mg/dL)	1.11 ± 0.79	1.05 ± 0.76	1.48 ± 0.90
Direct bilirubin (mg/dL)	0.83 ± 4.77	0.85 ± 5.14	0.71 ± 0.40
AST (U/L)	74.19 ± 127.23	74.55 ± 135.35	71.95 ± 54.40
ALT (U/L)			
ALP (U/L)	57.63 ± 76.76	58.55 ± 80.85	51.84 ± 43.29
Platelet (x103/µL)	131.08 ± 91.77	125.10 ± 90.44	167.74 ± 92.56
ALBI score	167.92 ± 88.73	173.18 ± 83.57	127.58 ± 109.37
mALBI grade	-2.38 ± 0.56	-2.44 ± 0.54	-1.96 ± 0.48
1			
2	120 (43.3%)	116 (48.5%)	4 (10.5%)
- 2a	142 (51.3%)	112 (46.9%)	30 (18.4%)
- 2b	- 48 (17.3%)	- 41 (17.2%)	- 7 (18.4%)
3	- 94 (33.9%)	- 71 (29.7%)	- 23 (60.5%)
	15 (5.4%)	11 (4.6%)	4 (10.5%)

ALBI, albumin-bilirubin; ALP, alkaline phosphatase; ALT, alanine aminotransferase; AST, aspartate aminotransferase; BCLC, Barcelona Clinical Liver Cancer; CTP, Child-Turcotte-Pugh; HBV, Hepatitis B virus; HCC, hepatocellular carcinoma; HCV, Hepatitis C virus; HRV, high-risk varices; mg/dL, milligram per deciliter; MASLD; Metabolic associated liver disease; ng/mL, nanogram per milliliter; PBC, primary biliary cirrhosis; PVT, portal vein thrombosis; SD, standard deviation; U/L, units per liter; µL, microliter.

### Comparison of characteristics between patients with and without HRV and factors associated with HRV in patients with HCC

Thirty-eight individuals (15.6%) were diagnosed with HRV (F2-3), 63 (22.7%) with small varices (F1), 176 (63.5%) with no varices on EGD, and 97 (35.0%) had portal hypertensive gastropathy. The proportion of patients with splenomegaly (81.6%) was significantly higher in the HRV group. Patients with HRV had lower albumin and platelet counts but higher total bilirubin levels (Table 1). Therefore, the mean ALBI score in patients with HRV is higher than that in those without HRV (–1.96 ± 0.48 vs. –2.44 ± 0.54).

On multivariate analysis, mALBI grades 2b and 3 and platelet count ≤ 150,000/µL were significantly associated with HRV with an odds ratio (OR) of 3.49 (95% confidence interval [CI]: 1.61–7.59) and 3.92 (95% CI: 1.62–9.48), respectively (Table 2).

**Table 2:** The univariate and multivariate analyses of factors associated with high-risk varices in patients with HCC.

Factors	Univariate analysis		Multivariate analysis	
	OR (95%CI)	p-value	OR (95%CI)	p-value
Male sex	1.10 (0.47-2.53)	0.832	-	
Age	0.99 (0.97-1.03)	0.888	-	
Viral hepatitis	0.50 (0.24-1.07)	0.075	-	
Splenomegaly	1.00 (1.00-1.00)	0.31	-	
CTP				
A	Ref.			
B	2.45 (1.00-5.99)	0.05	-	
BCLC stage				
A	Ref.			
B	1.32 (0.61-2.85)	0.475	-	
C	0.87 (0.34-2.22)	0.77	-	
PVT	1.24 (0.45-3.46)	0.679	-	
mALBI grade				
1 and 2a	Ref.		Ref.	
2b and 3	4.70 (2.22-9.95)	<0.001*	3.49 (1.61-7.59)	0.002*
platelet ≤ 150,000/µL	5.28 (2.24-12.47)	< 0.001*	3.92 (1.62-9.48)	0.002*

*p-value < 0.05.

BCLC, Barcelona Clinic Liver Cancer staging; CI, confidence interval; CTP, Child-Turcotte-Pugh; mALBI, modified albumin-bilirubin; OR, odds ratio, Ref.; reference.

### Performance of mALBI-PLT in predicting HRV in patients with HCC across training and validation cohorts, and through bootstrap internal validation

To assess the performance of the mALBI-PLT in predicting HRV, we used the first half of the participants as the training cohort (n = 138) and the second half as the validation cohort (n = 139). S1 Table presents the characteristics of the patients in the training and validation cohorts. Sixteen (11.6%) and twenty-two (15.8%) participants had HRV in the training and validation cohorts, respectively. S2 Table details the distribution of patients with different mALBI-PLT scores in the training and validation cohorts.

The area under the receiver operating characteristic curve (AUROC) of the mALBI-PLT score for predicting HRV in the training and validation cohort was 0.756 (95% CI: 0.648–0.834) and 0.743 (95% CI: 0.651–0.845), respectively. The mALBI-PLT at a cut-off of 2 exhibited similar performance in detecting HRV in the training and validation cohorts (Table 3). mALBI-PLT at a cut-off of 2 had a sensitivity of 93.8% (95% CI: 69.8–99.8) and an NPV of 98.1% (95% CI: 89.7–100) in the training cohort, and a sensitivity of 95.5%(95% CI: 77.2–99.9) and NPV of 98.0% (95% CI: 89.4–99.9) in the validation cohort.

**Table 3:** The sensitivity, specificity, positive predictive values, and negative predictive values of each cut-off of ALBI-PLT and mALBI-PLT scores for predicting HRV in the training and validation cohorts of patients with HCC.

Scores	Sensitivity	Specificity	PPV	NPV	LR+	LR-
**Training cohort (N = 138)**						
mALBI-PLT						
>2	93.8%	41.8%	17.4%	98.1%	1.61	0.15
	(69.8-99.8)	(32.9-51.1)	(10.1-27.1)	(89.7-100)	(1.32-1.96)	(0.02-1.01)
>3	62.5%	77.9%	27.0%	94.1%	2.82	0.48
	(35.4-84.8)	(69.5-84.9)	(13.8-44.1)	(87.5-97.8)	(1.7-4.68)	(0.25-0.91)
**Validation cohort (N = 139)**						
mALBI-PLT						
>2	95.5%	41.9%	23.6%	98.0%	1.64	0.11
	(77.2-99.9)	(32.8-51.4)	(15.2-33.8)	(89.4-99.9)	(1.37-1.96)	(0.02-0.75)
>3	54.5%	78.6%	32.4%	90.2%	2.55	0.58
	(32.2-75.6)	(70.1-85.7)	(18-49.8)	(82.7-95.2)	(1.52-4.28)	(0.36-0.92)

ALBI, albumin-bilirubin; ALBI-PLT, albumin-bilirubin and platelet; LR + , Likelihood ratios for positive results; LR-, likelihood ratios for negative results; mALBI, modified albumin-bilirubin; mALBI-PLT, modified ALBI-PLT; N, number; NPV, negative predictive value; PPV, positive predictive value.

Additionally, bootstrap resampling method was utilized to validate the mALBI-PLT score and its cut-off value two internally. The mALBI-PLT model demonstrated an apparent performance with a c-statistic of 0.748 (95% CI: 0.679–0.817). After bootstrap validation with 50 resamples, the c-statistic showed consistency at 0.749 (95% CI: 0.678–0.827). At its cut-off of two, the mALBI-PLT model displayed a notable performance with a c-statistic of 0.683 (95% CI: 0.635–0.731). Following bootstrap validation with 50 resamples, the c-statistic was 0.688 (95% CI: 0.651–0.751). The bootstrap performance demonstrated stable performance and acceptable discrimination of both mALBI-PLT score and its cut-off two.

### Validation of the ALBI-PLT score in the entire cohort and comparison with the mALBI-PLT score

**Fig 1:**
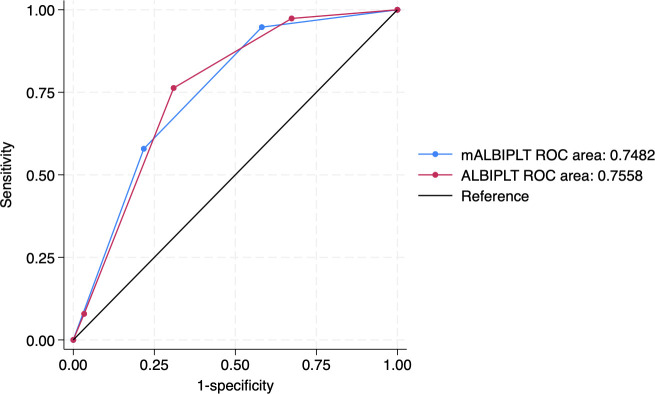
ROC of ALBI-PLT and mALBI-PLT score for predicting HRV in patients with HCC (p = 0.739). ALBI; albumin-bilirubin; HCC, hepatocellular carcinoma; HRV, high risk varices; ROC, receiver operating characteristic curve.

We validated the original ALBI-PLT using the entire cohort and compared its performance with the mALBI-PLT score. S3 Table illustrates the distribution of patients with HCC based on the ALBI-PLT and mALBI-PLT scores. Overall, 79 (28.5%) and 102 (36.8%) patients had an ALBI-PLT score of 2 and an mALBI-PLT score of 2, respectively. Furthermore, a significantly higher proportion of patients without HRV had ALBI-PLT and mALBI-PLT scores of 2. The AUROC of the mALBI grade and platelet count for predicting HRV was 0.721 (95%CI 0.648–0.794) and 0.738 (95% CI: 0.640–0.836), respectively (S2 Fig). The AUROC of ALBI-PLT was 0.756 (95% CI 0.691–0.821), and was non-significantly different from mALBI-PLT, which was 0.748 (95% CI 0.679–0.817, p = 0.739) (Fig 1).

The sensitivities of platelet ≤ 150,000/µL, ALBI grades 2 and 3, and mALBI score grades 2b and 3 for predicting HRV were 81.6% (95%CI 65.7–92.3), 89.5% (95%CI 75.2–97.1), and 71.1% (95%CI 54.1–84.6), respectively. Combining the ALBI or mALBI grade with the platelet count improved the sensitivity in identifying HRV, with ALBI-PLT > 2 and mALBI-PLT > 2 demonstrating sensitivities of 97.4% (95%CI 86.2–99.9) and 94.7% (95%CI 82.3–95.4), respectively. Additionally, ALBI-PLT > 2 and mALBI-PLT > 2 exhibited excellent NPV of 98.7% (95%CI 93.1–100) and 98.0% (95%CI 93.1–99.8), respectively (Table 4).

**Table 4:** The sensitivity, specificity, positive predictive values, and negative predictive values of each cut-off of ALBI-PLT and mALBI-PLT scores for predicting HRV in the entire cohort.

Scores	Sensitivity	Specificity	PPV	NPV	LR+	LR-
platelet ≤ 150,000/µL	81.6%	54.4%	22.1%	94.9%	1.79	0.34
	(65.7-92.3)	(47.8-60.8)	(15.6-29.9)	(89.8-97.9)	(1.46-2.2)	(0.17-0.67)
ALBI grade 2 and 3	89.5%	48.5%	21.7%	96.7%	1.74	0.22
	(75.2-97.1)	(42.0-55.1)	(15.5-28.9)	(91.7-99.1)	(1.47-2.05)	(0.09-0.55)
mALBI grade 2b and 3	71.1%	65.7%	24.8%	93.5%	2.07	0.44
	(54.1-84.6)	(59.3-71.7)	(17.0-34.0)	(88.6-96.7)	(1.58-2.71)	(0.27-0.73)
ALBI-PLT						
>2	97.4%	32.6%	18.7%	98.7%	1.45	0.08
	(86.2-99.9)	(26.7-39.0)	(13.5-24.8)	(93.1-100)	(1.30-1.60)	(0.01-0.56)
>3	76.3%	69.0%	28.2%	89.7%	2.46	0.34
	(59.8-88.6)	(62.8-74.8)	(19.7-37.9)	(90.4-97.6)	(1.90-3.19)	(0.193—0.61)
>4	7.89%	96.7%	27.3%	86.8%	2.36	0.95
	(1.66-21.4)	(93.5-98.5)	(6.02-61)	(82.2-90.7)	(0.65-8.5)	(0.87-1.05)
mALBI-PLT						
>2	94.7%	41.8%	20.6%	98.0%	1.63	0.13
	(82.3-95.4)	(35.3-48.4)	(14.8-27.3)	(93.1-99.8)	(1.43-1.86)	(0.03-0.49)
>3	57.9%	78.2%	29.7%	92.1%	2.66	0.54
	(40.8-73.7)	(72.5-83.3)	(19.7-41.5)	(87.5-95.4)	(1.85-3.82)	(0.37-0.79)

ALBI, albumin-bilirubin; ALBI-PLT, Albumin-bilirubin and platelet; LR + , Likelihoood ratios for positive results; LR-, likelihood ratios for negative results; mALBI, modified albumin-bilirubin; mALBI-PLT, modified ALBI-PLT; NPV, negative predictive value; PPV, positive predictive value.

When considering the negative likelihood ratio (LR-), individual parameters such as platelet count ≤ 150,000/µL, ALBI grades 2 and 3, and mALBI grades 2b and 3, yield LR- values of 0.34 (95%CI 0.17–0.67), 0.22 (95%CI 0.09–0.55), and 0.44 (95%CI 0.27–0.73), respectively, suggesting only a moderate ability to rule out HRV. In contrast, the composite scores ALBI-PLT and mALBI-PLT demonstrate noticeably better performance, with LR- values of 0.08 (95%CI 0.01–0.56) and 0.13 (95%CI 0.03–0.49), respectively, at a cut-off of 2. This indicates a strong ability to rule out HRV.

The EGD avoidance rate of mALBI-PLT 2 was significantly higher than that of ALBI-PLT 2 (36.8% vs. 28.5%, p = 0.001), whereas the false-negative rate was insignificant (5.3% vs. 2.6%, p = 0.317) (Table 5). In the subgroup analysis, the sensitivity and NPV of mALBI-PLT > 2 decreased in patients with non-viral hepatitis [sensitivity, 91.7% (95%CI 61.5–99.8); NPV, 94.4%(95%CI 72.7–99.9)] and BCLC C [sensitivity, 85.7% (95%CI 42.1–99.6); NPV, 95.8% (95%CI 78.9–99.9)]. In contrast, a subgroup of patients with viable HCC showed better sensitivity and NPV for predicting HRV [96.4% (95%CI 81.7–99.9) and 98.6%(95%CI 92.2–100)], respectively (S4 Table).

**Table 5:** The ratio of EGD avoidance and the missing HRV (entire cohort).

	ALBI-PLT = 2	mALBI-PLT = 2	p-value
EGD avoidance ratio (%)	79/277 (28.5%)	102/277 (36.8%)	<0.001*^¶^
False negative ratio (%)	1/38 (2.6%)	2/38 (5.3%)	0.317^¶^

*p-value < 0.05.

^¶^McNemar test.

ALBI, albumin-bilirubin; ALBI-PLT, Albumin-bilirubin and platelet; EGD, esophagogastroduodenoscopy; HRV, high-risk varices; mALBI, modified albumin-bilirubin; mALBI-PLT, modified ALBI-PLT.

### Patient survival within the cohort and factors influencing their survival

Among 271 patients who were dead or followed for 6 months or more, 12 (4.4%) experienced variceal bleeding, and 110 (40.6%) died. The causes of death were pulmonary embolism in 1 (0.9%), intracerebral hemorrhage in 1 (0.9%), EV bleeding in 2 (1.8%), decompensated liver disease in 4 (3.6%), ruptured hepatoma in 6 (5.5%), progression of HCC in 6 (5.5%), septic shock in 8 (7.3%), and unidentified cause of death in 80 (72.7%). The median survival after EGD of patients with HRV of 31.0 (95% CI: 16–not reached) months was significantly shorter than that of those without HRV of 93.0 (95% CI: 16–not reached, p = 0.017) months. The median survival of patients with ALBI-PLT scores of 2 and mALBI-PLT scores of 2 was not reached. In contrast, patients with ALBI-PLT > 2 and mALBI-PLT > 2 had a shorter median survival of 51.0 (95% CI: 37–93) months and 51.0 (95% CI: 38–59) months, respectively (S3 Fig). In the multivariate analysis, BCLC stages B and C, HRV, and mALBI-PLT > 2 were significantly associated with survival in patients with HCC with hazard ratios (HR) of 2.66 (95% CI: 1.64–4.34), 6.83 (95% CI: 4.15–11.24), 2.00 (95% CI: 1.19–3.38), and 1.96 (95% CI: 1.24–3.08), respectively (S5 Table).

## Discussion

The Baveno VII criteria use NITs, including LSM by TE and platelet count, to predict PHT and avoid endoscopic variceal screening in cirrhosis; however, evidence supporting its application in HCC is controversial and lacking [10–12]. Moreover, the tumor may influence LSM values, leading to unreliable measurements in patients with HCC [25]. Accordingly, this study focused on reproducible and objective markers for assessing liver function and portal hypertension; specifically, the ALBI score and platelet count [14,26]. These parameters have been combined as the ALBI-PLT score and demonstrated excellent performance for HRV prediction in patients with HCC [16]. Therefore, our study first validated the ALBI-PLT score and refined it for predicting HRV in patients with HCC.

In our study, the original ALBI-PLT at a cut-off of 2 demonstrated excellent sensitivity (97.4%) and NPV (98.7%). Our results are similar to those of initial studies by Chen et al., which found that an ALBI-PLT at a cut-off of 2 revealed an NPV of 97.1% and 98.1% in the study and validation cohorts, respectively, for ruling out varices in patients with HCC. [16] The NPV of 98.7% in our study aligns with that reported in the initial study by Chen et al. and confirms the excellent ability of the ALBI-PLT score to exclude HRV.

In the multivariable logistic regression analysis, mALBI grades 2b and 3, along with a platelet count ≤ 150,000/µL, demonstrated a significant association with HRV, with ORs of 3.49 (95%CI 1.61–7.59) and 3.92 (95%CI 1.62–9.48), respectively. Consequently, we developed a modified ALBI-PLT scoring system based on the beta coefficients derived from the multivariable logistic regression. In this scoring system, an mALBI-PLT score of 2 corresponds to patients with mALBI grade 1 or 2a and a platelet count > 150,000/µL. The mALBI-PLT score at a cut-off of 2 showed a similar outstanding performance in detecting HRV in the training and validation cohorts, with a sensitivity and NPV of approximately 95% and 98%, respectively. In addition, the bootstrap performance exhibited stable performance and acceptable discrimination of both mALBI-PLT score and its cut-off of 2, which supports the predictive utility of the mALBI-PLT score.

Of the entire cohort, an mALBI-PLT score >2 demonstrated higher sensitivity and NPV, along with a lower LR-, compared to mALBI grade 2b and 3, or platelet count > 150,000/µL alone. These findings suggest that combining mALBI grade and platelet count provides a superior discriminatory ability for ruling out HRV than either parameter used independently. In addition, the mALBI-PLT performed similarly to the ALBI-PLT, with a sensitivity of 94.7% and an NPV of 98.0%. Compared with the ALBI-PLT score, the mALBI-PLT significantly increased the rate of EGD avoidance, whereas the non-significant HRV ratio was missed.

Our cohort comprised 66.8% of patients with viable HCC and 35.7% with newly diagnosed HCC. Therefore, we performed a subgroup analysis in patients with viable HCC and confirmed that ALBI-PLT and mALBI-PLT at a cut-off of 2 demonstrated excellent sensitivity and NPV. Our study, reflecting real-world conditions with mixed treated and new cases, also discovered that ALBI-PLT and mALBI-PLT performed well. In a previous study validating the ALBI-PLT score in patients with cirrhosis, Inoue-Yuri et al. demonstrated impressive sensitivity and NPV rates of 98.2% and 98.6%, respectively [17]. Thus, ALBI-PLT and mALBI-PLT are likely reliable predictors of HRV in HCC and cirrhosis, thus extending their use to this mixed cohort of viable and non-viable HCC.

Furthermore, our study revealed a correlation between HRV, mALBI-PLT scores, and survival among patients with HCC. Consistent with a previous study, patients with HCC and HRV demonstrated a significantly reduced median survival of 31 months compared to 93 months in those without HRV [27]. Moreover, patients with HCC and mALBI-PLT > 2 showed a significantly shortened median survival of 51.0 (95% CI: 38–59) months than patients with mALBI-PLT 2 (median survival not reached). Additionally, mALBI-PLT > 2 was significantly related to survival with an HR of 1.96 (95% CI: 1.24–3.08). Our findings align with those of prior research that utilized a combination of albumin, total bilirubin, and platelet counts, demonstrating a predictive value for survival [28,29]. Incorporating platelet count into the ALBI score further indicates the severity of PHT and shows a stronger correlation with mortality in patients with HCC [28,29]. Our study confirms this concept and underscores the significance of PHT in patients with HCC.

This study is the first to validate ALBI-PLT in a real-world HCC clinic and employ a modified ALBI grade to predict HRV. Our findings support using the ALBI score with platelet counts to assess varices. Additionally, the components of ALBI-PLT and mALBI-PLT, including albumin level, total bilirubin level, and platelet count, are routine laboratory tests available even in resource-limited settings and do not require any further procedures or investigations. These advantages emphasize the simplicity and broad applicability of ALBI-PLT and mALBI-PLT for predicting HRV in patients with HCC [30].

However, the retrospective nature of our study poses some limitations. First, the indications for EGD in patients with HCC are not well established; therefore, not all patients with HCC in our clinic underwent EGD for variceal screening, and were excluded from the studies. Future prospective studies with more comprehensive data collection are required to minimize selection bias. Second, our study participants were patients without cirrhosis and those with cirrhosis CTP A or B. Therefore, applying the ALBI-PLT and mALBI-PLT scores in patients with HCC with decompensated cirrhosis (CTP C) requires further validation. Third, measuring TE in HCC is challenging due to the mass effect and lack of a TE cut-off for clinical application; only 26 (9.4%) patients with TE values were enrolled in our study. Therefore, we did not directly compare the Baveno VII criteria with mALBI-PLT for predicting HRV because of the limited number of patients with TE in our study. Future studies should consider direct comparisons with TE or explore the combination of TE or other imaging modalities with ALBI-PLT or mALBI-PLT to enhance predictive accuracy. In addition, PVT was found in 26 out of 239 (10.9%) participants with HRV and in 5 out of 38 (13.2%) participants without HRV. However, in the univariate analysis, PVT demonstrated a non-significant OR of 1.24 (95%CI: 0.45–3.46) for predicting HRV. The results of our study differ from those of previous studies, which indicated a significant association between PVT—particularly main portal vein involvement—and HRV [31,32]. This discrepancy may result from the heterogeneity among enrolled HCC patients, including the inclusion of patients with previously treated HCC (inactive HCC), and the small number of patients with PVT in our study. A larger cohort may be necessary to clarify the impact of PVT on the development of HRV in patients with HCC. Finally, deaths were identified from the Thailand Civil Registration Database, which does not record the cause of death. This finding prevented us from investigating the association between death and variceal bleeding.

## Conclusion

In conclusion, our study is the first to validate the performance of the ALBI-PLT score for HRV prediction in patients with HCC. Additionally, we demonstrated a significant association between mALBI grades 2b and 3 and HRV and proposed a refined mALBI-PLT score for HRV prediction. The mALBI-PLT score at a cut-off of 2 revealed excellent sensitivity and NPV of 94.7% and 98.0%, respectively, for HRV prediction and an acceptable HRV missing rate of 5.3%. Therefore, patients with HCC and mALBI grade 1, which may include grade 2a, and a platelet count > 150,000/µL may be able to avoid EGD.

## Supporting information

S1 Fig Flowchart of patient eligibility. (DOCX)

S2 Fig ROC of mALBI grade and platelet count for predicting HRV in patients with HCC. (DOCX)

S3 Fig Kaplan-Meier curves showing survival probability according to (A) presence of HRV, (B) ALBI-PLT > 2 vs ALBI-PLT = 2,and (C) ALBI-PLT > 2 vs ALBI-PLT = 2. (DOCX)

S1 Table Demographic, clinical, and tumor characteristics and laboratory values of patients in the training and validation cohort. (DOCX)

S2 Table ALBI-PLT and mALBI-PLT scores in HCC patients with and without HRV (training and validation cohort). (DOCX)

S3 Table ALBI-PLT and mALBI-PLT scores in HCC patients with and without HRV (entire cohort). (DOCX)

S4 Table The sensitivity, specificity, positive predictive values, and negative predictive values of ALBI-PLT > 2 and mALBI-PLT > 2 in subgroup analysis to predict HRV. (DOCX)

S5 Table The uni- and multivariate analysis of factors associated with survival in patients with HCC using cox regression. (DOCX)

S1 File Raw data. (XLSX)
