# Supplementary material for: Combination of modified albumin-bilirubin grade and platelet count to predict high-risk varices in patients with hepatocellular carcinoma
Source: PLoS One. 2025 Jul 17;20(7):e0327967. doi: 10.1371/journal.pone.0327967 (PMC12270117; doi:10.1371/journal.pone.0327967)
Supplement: S1 Fig — (DOCX) [file pone.0327967.s001.docx]

**Supplementary Figure 1** Flowchart of patient eligibility


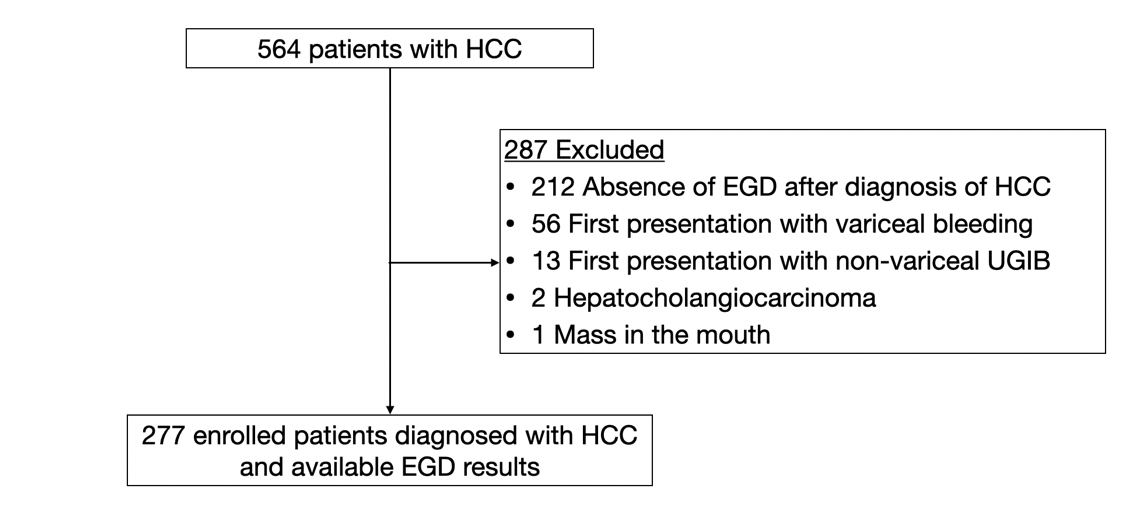


EGD; Esophagogastroduodenoscopy, HCC; Hepatocellular carcinoma, N; Number, UGIB; Upper gastrointestinal bleeding.
