# Supplementary material for: Combination of modified albumin-bilirubin grade and platelet count to predict high-risk varices in patients with hepatocellular carcinoma
Source: PLoS One. 2025 Jul 17;20(7):e0327967. doi: 10.1371/journal.pone.0327967 (PMC12270117; doi:10.1371/journal.pone.0327967)
Supplement: S2 Fig — (DOCX) [file pone.0327967.s002.docx]

**Supplementary figure 2** ROC of mALBI grade and platelet count for predicting HRV in patients with HCC

| **mALBI grade** | **Platelet count** |
| --- | --- |
|  |  |
| AUROC 0.721 (0.648-0.794) | AUROC 0.738 (0.640-0.836) |

mALBI; modified albumin-bilirubin; AUROC, area under receiver operating characteristic curve; HCC, hepatocellular carcinoma; HRV, high risk varices; ROC, receiver operating characteristic curve
