# Supplementary material for: Combination of modified albumin-bilirubin grade and platelet count to predict high-risk varices in patients with hepatocellular carcinoma
Source: PLoS One. 2025 Jul 17;20(7):e0327967. doi: 10.1371/journal.pone.0327967 (PMC12270117; doi:10.1371/journal.pone.0327967)
Supplement: S3 Fig — (DOCX) [file pone.0327967.s003.docx]

**Supplementary Figure 3** Kaplan-Meier curves showing survival probability according to (A) presence of HRV, (B) ALBI-PLT >2 vs ALBI-PLT = 2 ,and (C) ALBI-PLT >2 vs ALBI-PLT = 2.

| **A** (p 0.017) | |
| --- | --- |
| 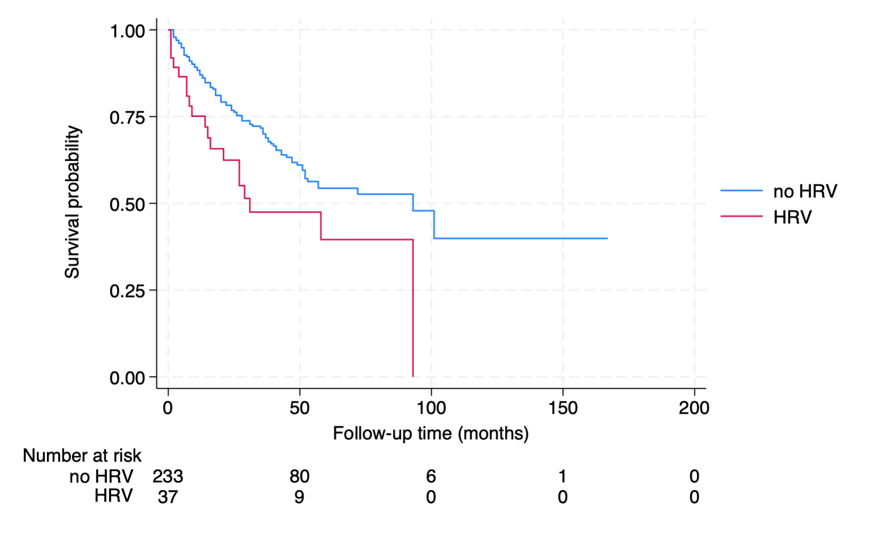 | |
| **B** (p < 0.001) | **C** (p 0.005) |
| 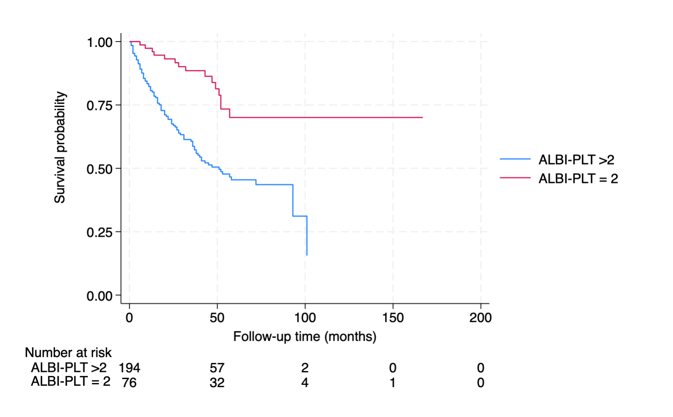 | 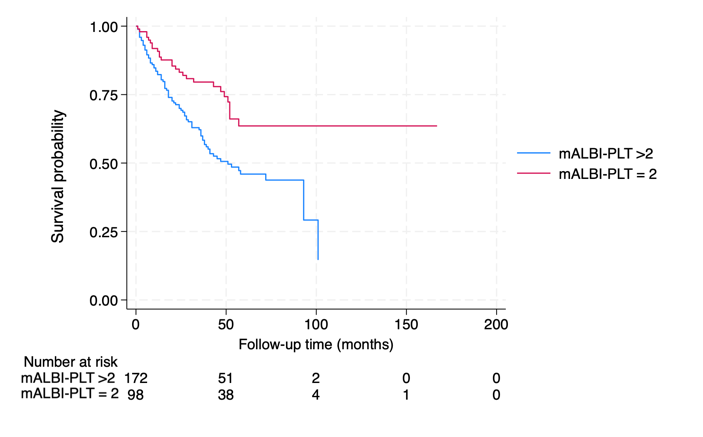 |

ALBI-PLT, Albumin-bilirubin and platelet; HCC, hepatocellular carcinoma; HRV, high-risk varices; mALBI-PLT, modified ALBI-PLT.
