# Supplementary material for: Combination of modified albumin-bilirubin grade and platelet count to predict high-risk varices in patients with hepatocellular carcinoma
Source: PLoS One. 2025 Jul 17;20(7):e0327967. doi: 10.1371/journal.pone.0327967 (PMC12270117; doi:10.1371/journal.pone.0327967)
Supplement: S1 Table — (DOCX) [file pone.0327967.s004.docx]

**Supplementary Table 1** Demographic, clinical, and tumor characteristics and laboratory values of patients in the training and validation cohort

| Characteristics  **Number (%)/ mean ± SD** | **Training cohort**  **(N = 138)** | **Validation cohort**  **(N= 139)** | **p-value** |
| --- | --- | --- | --- |
| Male  Age  Etiologies of cirrhosis  HBV  HCV  HBV and HCV  Alcohol  MASLD  Cryptogenic  PBC  Cirrhosis  CTP  A (5-6)  B (7-8)  Splenomegaly  BCLC staging  A  B  C  - PVT  Viable HCC  Yes  No  Liver function tests  Albumin (g/dL)  Total bilirubin (mg/dL)  Direct bilirubin (mg/dL)  AST (U/L)  ALT (U/L) ALP (U/L)  Platelet (x10^3^/µL)  ALBI score  mALBI grade  1  2  - 2a  - 2b  3 | 104 (75.4%)  69.78 ± 10.17  71 (51.4%)  45 (32.6%)  1 (0.7%)  10 (7.2%)  4 (2.9%)  6 (4.3%)  1 (0.7%)  131 (94.9%)  121 (87.7%)  10 (7.2%)  63 (45.7%)  80 (58.0%)  43 (31.2%)  15 (10.9%)  - 8 (5.8%)  78 (56.5%)  60 (43.5%)  3.82 ± 0.48  1.1092 ±63.95 ± 0.81  0.49 ± 0.40  64.28 ± 79.96  57.61 ± 66.78  112.83 ± 52.26  147.92 ± 63.95  -2.46 ± .51  67 (48.6%)  68 (49.3%)   - 28 (20.3%) - 40 (29.0%)   3 (2.2%) | 111 (79.9%)  63.09 ± 11.37  63 (45.3%)  39 (28.1%)  1 (0.7%)  17 (12.2%)  7 (5.0%)  12 (8.6%)  0 (0%)  131 (94.2%)  111 (79.9%)  20 (14.4%)  78 (56.1%)  51 (36.7%)  42 (40.2%)  46 (33.1%)  - 23 (16.5%)  107 (77.0%)  32 (23.0%)  3.63 ± 0.60  1.12 ± 0.77  1.16 ± 6.73  83.96 ± 160.50  57.64 ± 85.70  148.67 ± 115.46  185.79 ± 104.72  -2.29 ± 0.59  53 (38.1%)  74 (53.2%)   - 20 (14.4%) - 54 (38.8%)   12 (8.6%) | 0.370^†^  0.322^‡^  0.802^†^  0.052^†^  0.196^†^  **<0.001^†^**  **<0.001^†^**  **0.009^$^**  0.331^$^  **0.045^$^**  **0.078^$^**  0.747^$^  **0.006^$^**  **0.009^$^**  **0.012^$^**  **0.015^†^** |

^†^Pearson Chi-Square

^‡^Fischer exact test

^$^Mann-Whitney U

ALBI, albumin-bilirubin; ALP, alkaline phosphatase; ALT, alanine aminotransferase; AST, aspartate aminotransferase; BCLC, Barcelona Clinical Liver Cancer; CTP, Child-Turcotte-Pugh; HBV, Hepatitis B virus; HCC, hepatocellular carcinoma; HCV, Hepatitis C virus; HRV, high-risk varices; mg/dL, milligram per deciliter; MASLD; Metabolic associated liver disease; ng/mL, nanogram per milliliter; PBC, primary biliary cirrhosis; PVT, portal vein thrombosis; SD, standard deviation; U/L, units per liter; µL, microliter
