# Supplementary material for: Combination of modified albumin-bilirubin grade and platelet count to predict high-risk varices in patients with hepatocellular carcinoma
Source: PLoS One. 2025 Jul 17;20(7):e0327967. doi: 10.1371/journal.pone.0327967 (PMC12270117; doi:10.1371/journal.pone.0327967)
Supplement: S2 Table — (DOCX) [file pone.0327967.s005.docx]

**Supplementary Table 2** ALBI-PLT and mALBI-PLT scores in HCC patients with and without HRV (training and validation cohort)

|  | **Training cohort**  **(N = 138)** | **No HRV**  **(N = 122)** | **HRV**  **(N = 16)** | **p-value** |
| --- | --- | --- | --- | --- |
| mALBI-PLT  2  3  4 | 52 (37.7%)  49 (35.5%)  37 (26.8%) | 51 (41.8%)  44 (36.1%)  27 (22.1%) | 1 (6.3%)  5 (31.3%)  10 (62.5%) | 0.001*^†^ |
|  | **Validation cohort**  **(N = 139)** | **No HRV**  **(N = 117)** | **HRV**  **(N = 22)** | **p-value** |
| mALBI-PLT  2  3  4 | 50 (36.0%)  52 (37.4%)  37 (26.6%) | 49 (41.9%)  43 (36.8%)  25 (21.4%) | 1 (4.5%)  9 (40.9%)  12 (54.5%) | < 0.001*^†^ |

*Significant p-value ≤ 0.05

^†^Pearson Chi-Square

ALBI-PLT, Albumin-bilirubin and platelet; HRV, high-risk varices; mALBI-PLT, modified ALBI-PLT.
