# Supplementary material for: Combination of modified albumin-bilirubin grade and platelet count to predict high-risk varices in patients with hepatocellular carcinoma
Source: PLoS One. 2025 Jul 17;20(7):e0327967. doi: 10.1371/journal.pone.0327967 (PMC12270117; doi:10.1371/journal.pone.0327967)
Supplement: S3 Table — (DOCX) [file pone.0327967.s006.docx]

**Supplementary Table 3** ALBI-PLT and mALBI-PLT scores in HCC patients with and without HRV (entire cohort)

|  | **All patients**  **(N = 277)** | **No HRV**  **(N= 239)** | **HRV**  **(N=38)** | **p-value** |
| --- | --- | --- | --- | --- |
| ALBI-PLT  2  3  4  5 | 79 (28.5%)  95 (34.3%)  92 (33.2%)  11 (4.0%) | 78 (32.6%)  87 (36.4%)  66 (27.6%)  8 (3.3%) | 1 (2.6%)  8 (21.1%)  26 (68.4%)  3 (7.9%) | <0.001*^†^ |
| mALBI-PLT  2  3  4 | 102 (36.8%)  101 (36.5%)  74 (26.7%) | 100 (41.8%)  87 (36.4%)  52 (21.8%) | 2 (5.3%)  14 (36.8%)  22 (57.9%) | <0.001*^†^ |

*Significant p-value ≤ 0.05

^†^Pearson Chi-Square

ALBI-PLT, Albumin-bilirubin and platelet; HRV, high-risk varices; mALBI-PLT, modified ALBI-PLT.
