# Supplementary material for: Combination of modified albumin-bilirubin grade and platelet count to predict high-risk varices in patients with hepatocellular carcinoma
Source: PLoS One. 2025 Jul 17;20(7):e0327967. doi: 10.1371/journal.pone.0327967 (PMC12270117; doi:10.1371/journal.pone.0327967)
Supplement: S4 Table — (DOCX) [file pone.0327967.s007.docx]

**Supplementary Table 4** The sensitivity, specificity, positive predictive values, and negative predictive values of ALBI-PLT >2 and mALBI-PLT >2 in subgroup analysis to predict HRV.

| **Subgroup** | **Scores** | **Sensitivity** | **Specificity** | **PPV** | **NPV** | **LR+** | **LR-** |
| --- | --- | --- | --- | --- | --- | --- | --- |
| Viral hepatitis  N = 220 (79.4%) | ALBI-PLT > 2  mALBI-PLT >2 | 96.2%  (80.4-99.9)  96.2%  (80.4-99.9) | 35.1%  (28.4-42.2)  42.8%  (35.7-50.1) | 16.6%  (11-23.5)  18.4%  (12.3-25.9) | 98.6%  (92.2-100)  98.8%  (93.5-100) | 1.48  (1.3-1.68)  1.68  (1.46-1.94) | 0.11  (0.159-0.757)  0.0899  (0.0131-0.619) |
| Non-viral hepatitis  N = 57 (20.6%) | ALBI-PLT > 2  mALBI-PLT >2 | 100%  (73.5-100)  91.7%  (61.5-99.8) | 22.2%  (11.2-37.1)  37.8%  (23.8-53.5) | 25.5%  (13.9-40.3)  28.2%  (15-44.9) | 100%  (69.2-100)  94.4%  (72.7-99.9) | 1.29  (1.1-1.5)  1.47  (1.11-1.96) | 0  0.221  (0.0325-1.5) |
| BCLC A,B  N = 216 (78.0%) | ALBI-PLT > 2  mALBI-PLT >2 | 96.8%  (83.3-99.9)  96.8%  (83.3-99.9) | 33.5%  (26.8-40.8)  41.6%  (34.4-49.1) | 19.6%  (13.6-26.8)  21.7%  (15.2-29.6) | 98.4%  (91.5-100)  98.7%  (93.1-100) | 1.46  (1.29-1.64)  1.66  (1.44-1.9) | 0.0963  (0.0138-0.669)  0.0775  (0.0112-0.537) |
| BCLC C  N = 61 (22.0%) | ALBI-PLT > 2  mALBI-PLT >2 | 100%  (59-100)  85.7%  (42.1-99.6) | 29.6%  (18-43.6)  42.6%  (29.2-56.8) | 15.6%  (6.49-29.5)  16.2%  (6.19-32) | 100%  (79.4-100)  95.8%  (78.9-99.9) | 1.42  (1.2-1.69)  1.49  (1.02-2.18) | 0  0.335  (0.532-2.11) |
| Non-viable HCC  N = 92 (33.2%) | ALBI-PLT > 2  mALBI-PLT >2 | 90.0%  (55.5-99.7)  90.0%  (55.5-99.7) | 32.9%  (22.9-44.2)  39.0%  (28.4-50.4) | 14.1%  (6.64-25)  15.3%  (7.22-27) | 96.4%  (81.7-99.9)  97.0%  (84.2-99.9) | 1.34  (1.04-1.73)  1.48  (1.13-1.93) | 0.304  (0.461-2)  0.256  (0.0391-1.68) |
| Viable HCC  N = 185 (66.8%) | ALBI-PLT > 2  mALBI-PLT >2 | 100%  (87.7-100)  96.4%  (81.7-99.9) | 32.5%  (25.2-40.4)  43.3%  (35.4-51.4) | 20.9%  (14.4-28.8)  23.3%  (15.9-32) | 100%  (93.0-100)  98.6%  (92.2-100) | 1.48  (1.33-1.65)  1.7  (1.46-1.98) | 0  0.0825  (0.0119-0.57) |

ALBI-PLT, Albumin-bilirubin and platelet; BCLC, Barcelona Clinical Liver Cancer; HCC, hepatocellular carcinoma; HRV, high risk varices; mALBI-PLT, modified ALBI-PLT; N, number; NPV, negative predictive value; PPV, positive predictive value; µL, microliter.
