# Supplementary material for: Combination of modified albumin-bilirubin grade and platelet count to predict high-risk varices in patients with hepatocellular carcinoma
Source: PLoS One. 2025 Jul 17;20(7):e0327967. doi: 10.1371/journal.pone.0327967 (PMC12270117; doi:10.1371/journal.pone.0327967)
Supplement: S5 Table — (DOCX) [file pone.0327967.s008.docx]

**Supplementary Table 5** The uni- and multivariate analysis of factors associated with survival in patients with HCC using cox regression

| **Factors** | **Univariate analysis** | | **Multivariate analysis** | |
| --- | --- | --- | --- | --- |
|  | **HR (95%CI)** | **p-value** | **HR (95%CI)** | **p-value** |
| Male sex  Age  Viral hepatitis  BCLC stage  A  B  C  HRV  mALBI-PLT > 2 | 0.81 (0.53-1.24)  0.98 (0.96-0.99)  0.65 (0.42-1.01)  Ref.  2.73 (1.69-4.43)  6.17 (3.81-9.99)  1.81 (1.10-2.97)  2.09 (1.35-3.23) | 0.337  0.035*  0.055  < 0.001*  < 0.001*  0.002*  0.001* | 0.99 (0.97-1.00)  -  Ref.  2.66 (1.64-4.34)  6.83 (4.15-11.24)  2.00 (1.19-3.38)  1.96 (1.24-3.08) | 0.144  -  < 0.001*  < 0.001*  0.009*  0.004* |

*p-value < 0.05

BCLC, Barcelona Clinic Liver Cancer staging; CI, confident interval; HRV, high-risk varices; mALBI-PLT, modified ALBI-PLT; HR, hazard ratio, Ref.; reference.
